# Examining the Relationship Between Incarceration and Healthy Aging

**DOI:** 10.1007/s40865-025-00286-5

**Published:** 2025-12-03

**Authors:** Elaine Eggleston Doherty, Brittany A. Bugbee, Kerry M. Green

**Affiliations:** Department of Behavioral and Community Health, University of Maryland, 4200 Valley Drive, College Park, MD 20742 USA

**Keywords:** Healthy aging, Life course, Longitudinal, Criminal legal system contact

## Abstract

Our understanding of the collateral consequences of incarceration on health, biological aging, and mortality has increased exponentially in recent years. Drawing on newly collected data on aging at age 62 and retrospective reports of incarceration history among a community cohort of Black men and women who have been prospectively followed from first grade (in 1966) to later life (modal age 62), this study adds to this growing literature by examining whether incarceration is associated with healthy aging, a concept that captures the aging experience through traditional indicators, such as physical and mental health conditions, as well as through indicators of functional ability and well-being, such as cognitive functioning, sleep, and hearing loss. By focusing on a first-grade, single-race cohort from the same socially-disadvantaged neighborhood, this study holds constant race, age, and early life context by design. Although largely a cross-sectional study, we also include key early life control variables in multivariable models. Results show that incarceration history is associated with less healthy aging compared to never being arrested using a global index and across many of the individual indicators. One unexpected finding is that many of these detriments are equally felt among the incarcerated and those with earlier stages of system contact (i.e., those arrested but not incarcerated). Taken together, these findings represent a first step in building scholarship on the association between criminal legal system contact and healthy aging, broadly defined, from a life course perspective and provide direction for future research in this emerging area.

**Supplementary Information:**

The online version contains supplementary material available at 10.1007/s40865-025-00286-5.

## Introduction

The experience of being incarcerated—being isolated from one’s community, stripped of one’s privacy and autonomy, and forced to adapt to a different set of rules and norms (e.g., Goffman, 1961)—shapes one’s life course across myriad dimensions. Indeed, the “pains” of imprisonment on the individual can lead to chronic stress, frustration, and reduced social well-being while incarcerated and long after release (Sykes, 1958; Irwin, 1985; Haney, 2003; Turney & Conner, 2019). Although the relationship between incarceration history and health is confounded by pre-existing conditions and varies in strength by health condition (see Schnittker et al., 2022), a growing body of research shows that experiences of incarceration can induce psychological distress, disrupt social relationships, and exacerbate economic disadvantage with further impacts on one’s psychological and physical health, biological aging, and mortality (e.g., Travis et al., 2014; Massoglia & Pridemore, 2015; Beckett & Goldberg, 2022; Schnittker et al., 2022; Sugie & Turney, 2017; Massoglia, 2008; Latham-Mintus et al., 2023; Garcia-Grossman et al., 2023; Berg et al., 2021, 2022; Bovell-Ammon et al., 2021; Massoglia et al., 2014). This study adds to the literature by examining whether incarceration is associated with healthy aging, a concept that encompasses more than disorders and conditions and captures the aging experience more broadly by incorporating traditional indicators, such as physical and mental health conditions, along with indicators of capabilities and well-being, such as physical and emotional functional limitations (World Health Organization, 2015).

Whereas aging can be biologically defined as the gradual yet “persistent decline in the age-specific fitness components of an organism due to internal physiological deterioration” (Rose, 1995: 20), this definition is narrow in its focus. For many, aging comes with disease and declining health, yet the term “healthy” aging connotes a more holistic multidimensional concept that incorporates not only a lack of disease but also a lack of cognitive impairment, sensory impairment (e.g., loss of vision or hearing), and social and psychosocial detriments (e.g., loneliness). According to the World Health Organization, healthy aging encompasses being free of disease and disability as well as “the process of developing and maintaining the functional ability that enables well-being in older age” (WHO, 2015, p. 28). Thus, a holistic operational definition of healthy aging would include a host of health and health-related indicators, such as physical, psychological, and cognitive capabilities and functioning along with the absence of disease (Cosco et al., 2014; Lu et al., 2019; Michel & Sadana, 2017; Zamudio-Rodríguez et al., 2021). In this study, we ask: Is incarceration history related to healthy aging, using a holistic definition of healthy aging?

An examination of incarceration and healthy aging is a particularly relevant line of inquiry in life course criminology given that: (1) the demographics of the US population have shifted such that, in 2020, people ages 65 or older comprised 17% of the US population, a 39% increase from 2010 (Caplan & Rabe, 2023); and (2) today’s 65-year-olds lived through the height of mass incarceration during their young adult lives. Each year between 1985 and 1995, the prison population increased 8% on average (Nellis, 2024).

A focus on the relationship between incarceration and healthy aging among Black Americans in their 60 s, which we do here, is particularly imperative for several reasons. First, the policies driving mass incarceration have been disproportionately felt by Black US adults. The risk of imprisonment for Black males rose from 20% in 1986 to 50% in 2004 (Roehrkasse & Wildeman, 2022); in 2002, Black Americans represented 12% of the US population but 43% of pretrial detainees in local jails (Sawyer, 2020); and in 2019, Black adults represented approximately 45% of the prison population serving 10 years or longer (Nellis, 2024). Second, racial disparities in mortality persist. Black Americans have a life expectancy close to six years shorter than white Americans (70.8 vs. 76.4; Andrasfay & Goldman, 2021, 2022; Arias et al., 2023), which translates into not only one’s own mortality risk but also the disproportionate exposure to the premature death of family and household members (Umberson et al., 2017; Dixon, 2024), which may shape one’s own aging trajectory. Third, the impacts of incarceration in shaping life chances—educational deficits, poor labor market experiences, strained social relationships, and system avoidance—are more extensively felt among Black Americans with an incarceration history than white Americans (Blankenship et al., 2018), which could translate into disparities in aging outcomes in later life.

### Incarceration on Domains of Aging

Research has repeatedly found a significant relationship between incarceration history and a variety of physical and mental health outcomes among young adults (e.g., 30-year-olds) (e.g., Boen, 2020; Porter & DeMarco, 2019), as well as older adults (e.g., 50-year-olds) (Latham-Mintus et al., 2023; Garcia-Grossman et al., 2023). Among older adults, several studies using nationally representative data (e.g., the Health Retirement Survey, the National Longitudinal Survey of Youth 1979) show that for many health-related outcomes, such as chronic physical and mental health conditions, incarceration history is associated with poorer health (e.g., Garcia-Grossmen et al., 2023), more physical limitations (Latham-Mintus et al., 2023), higher rates of functional impairments, such as hearing (Greene et al., 2018), and more cognitive impairments (Cox & Wallace, 2022).

Yet, incarceration is not universally related to all health and aging outcomes or for all populations, and its relationship is often subject to selection bias. Massoglia (2008) finds a stronger relationship between incarceration and stress-related physical conditions, such as hypertension and chronic lung disease, with no significant relationship found with non-stress-related conditions, such as cancer. Schnittker et al. (2012) find a robust relationship between incarceration and mood disorders but less so for other psychiatric disorders. Bovell-Ammon et al. (2021) find a relationship between incarceration and mortality for Black adults but not non-Black adults, and Cox and Wallace (2022) find that the relationship between incarceration and cognitive impairment largely disappears once key covariates are added to the statistical model (see Schnittker et al., 2022).

The predominant mechanism linking incarceration with physical and mental health is that incarceration acts as a primary stressor that weakens one’s resilience and compromises one’s physical and mental state (Pearlin, 1989; Massoglia, 2008; Sugie & Turney, 2017). Among Black Americans the differential exposure to incarceration represents one of the many by-products of structural racism that results in a higher likelihood in experiencing this stress and deterioration of health at the cellular level, a process known as weathering (Geronimus, 2023). Incarceration experiences also trigger secondary stressors (Pearlin, 1989) in the face of post-incarceration challenges, such as housing and social support. For instance, significant challenges stem from criminal legal system policies and stigma that can lead to blocked employment, housing, and educational opportunities thwarting upward mobility and threatening economic security (e.g., Sampson & Laub, 1997; Pager, 2008; Evans et al., 2019; Stewart & Uggen, 2020). The disruption of social bonds and social disengagement during incarceration can lead to a lack of social support and loneliness post-release (Laub & Sampson, 2003; Fader, 2021) as well as a distrust of others, hypervigilance, and social anxiety (Liem & Kunst, 2013; Schnittker, 2014; Hulley et al., 2016). Thus, the mechanisms underpinning the relationship between incarceration and the holistically-conceived definition of healthy aging are grounded in both the primary stressor of the incarceration experience as well as the numerous structural and interpersonal secondary stressors experienced during reintegration (Schnittker et al., 2022), including an inability to meet one’s basic needs through reduced financial security and a compromised ability to build and maintain relationships that in turn compromise healthy aging (WHO, 2015).

### Dimensions of Incarceration History and Healthy Aging

The bulk of the research examining the relationship between incarceration and health-related outcomes compare incarcerated groups to non-incarcerated groups. However, these comparisons do not adequately isolate the unique impact of incarceration separate from earlier stages of criminal legal system contact (Massoglia & Pridemore, 2015), such as arrest, which has also been found to have health consequences (Sugie & Turney, 2017; Doherty et al., 2016, 2022). Thus, when studying the consequences of incarceration, the recommendation is to compare an incarceration group with at least two comparison groups—those who have never been arrested and those who have been arrested but have never been incarcerated—to better isolate the experience of incarceration^1^ (Massoglia & Pridemore, 2015).

The number and dosage of incarceration histories have been found to be important considerations for health outcomes, such as mortality (Patterson, 2013) and mental health (Porter & DeMarco, 2019). Given that the experience of incarceration itself is not necessarily a universal one (e.g., short stays in jails versus long stays in prisons; Turney & Conner, 2019), the number and/or length of incarceration are important considerations when studying incarceration and healthy aging; yet, the direction of this distinction on the broad range of healthy aging indicators as well as their confluence is an open question. For instance, one might hypothesize that spending several consecutive years incarcerated creates more of a detrimental impact on one’s aging than even multiple short stays as a result of pre-existing mental health-related conditions that are likely to go untreated and likely to be exacerbated while in prison (Schnittker et al., 2022). One’s adaptation to prison life that doesn’t align with societal norms outside of prison may also hinder the aging process (Haney, 2003). Alternatively, one might hypothesize that multiple short-term stays marked by recurrent detention and release in and out of a jail setting can lead to strained relationships (e.g., repeated disappointments of family), financial hardships (e.g., requests for financial assistance, such as bail), and anticipatory stress that are more acutely felt than after a single, albeit long, incarceration in a prison setting (Sykes, 1958; Pogrebin et al., 2001; Turney & Conner, 2019; Turney et al., 2024).

### Current Study

This study adds to the life course consequences of incarceration literature by examining its relationship with healthy aging, conceptualized across multiple domains, including physical and physiological health (e.g., chronic pain, physical health conditions, functional limitations), psychological and social health (e.g., mental health conditions, functional limitations, sleep functioning, loneliness), and cognitive health (e.g., cognitive functioning, memory, hearing loss). Importantly, we examine these relationships among a community cohort of Black men and women who have been prospectively followed since first grade (in 1966) to later life (modal age 62) to inform how incarceration relates to aging among this highly relevant population. By focusing on a first-grade single-race cohort from the same socially-disadvantaged neighborhood, this study holds constant race, age, and early life context by design. Thus, the never arrested comparison group is similar to the justice-involved groups with respect to age, race, and early neighborhood context.

## Data and Methods

### Woodlawn Study

Data are drawn from the Woodlawn Study community cohort, which is a cohort of 1,242 men (48.8%) and women (51.2%) who attended first grade in 1966-67 in one of the 12 public and parochial schools in Woodlawn, a neighborhood in Chicago, Illinois (only 13 families declined to participate). In the 1960s, Woodlawn was a predominantly African American community (98%; Kellam et al., 1975) and was one of the most socially disadvantaged communities in Chicago at the time (Doherty & Green, 2023). The vast majority of the cohort were born in 1959 (13.9%) or 1960 (85.4%), identify as Black or African American (99.7%), and all lived in the same community in first grade.

The historical context from first grade to adulthood for this cohort is particularly relevant to their incarceration and health. For instance, the HIV/AIDS and crack epidemics began in the 1980s, when the cohort members were in their 20s; rates of gun violence, mass incarceration, and the expanded removal of social safety nets for those with felony convictions reentering the community sharply increased during their 30s (i.e., 1990s), and Medicaid exclusion policies were in place that created barriers for re-enrollment upon reentry. These contexts were coupled with the introduction of welfare reform during their 30s and the Affordable Care Act during their 40s, both of which enabled families to meet their basic needs and broadened access to health care.

The Woodlawn Study has captured prospective data spanning much of the cohort’s life course (ages 6 to 62). In first grade, teachers and mothers (or mother surrogates) reported on key domains, such as the family’s social and economic resources as well as the psychological and behavioral aspects of the child (Kellam et al., 1975). In adolescence, the mothers (*n* = 939) and adolescents (*n* = 705) living in the Chicago area provided extensive information on the family, behaviors, and psychological well-being (Ensminger & Slusarcick, 1992). Across adulthood, the original cohort was interviewed about their families, community involvement, drug and criminal behavior, criminal legal system involvement, and physical and mental health - in young adulthood (age 32, 1992–1993, *n* = 952), in midlife (age 42, 2002–2003, *n* = 833), and most recently in later life (age 62, 2022–2023, *n* = 531). The most recent interview (age 62) also included several measures tapping into experiences related to aging. For this study, we focus on the 531 participants (58.0% women and 42.0% men) who completed the age 62 interview to examine the relationship between retrospective accounts of incarceration history and current indicators of healthy aging among those who survived to their 60s. We use measures from the age 6 and adolescent assessments to control for early life confounders.

### Characterizing the Analytic Sample: Mortality and Attrition

One key consideration when studying incarceration and healthy aging among these 531 participants is the acknowledgement that close to one-quarter of the full first-grade cohort had passed away by 2023. Given evidence that incarceration increases the risk of mortality for Black adults, in particular (e.g., Bovell-Ammon et al., 2021), we estimated Kaplan-Meier curves and Cox regression models to assess mortality^2^ risk by incarceration and arrest history by age 42^3^ among the subset of the cohort with this information (*n* = 944; 26.9% incarcerated by age 42; see Appendix A). The results show that 24.0% of those incarcerated and 22.6% of those arrested but not incarcerated by age 42 died by 2023 (modal age 63) compared with 11.8% of the never arrested group. Against this backdrop, the research question becomes, is incarceration history related to healthy aging, *conditional* on surviving to age 62?

Another key consideration when studying incarceration and healthy aging among these 531 participants interviewed at age 62 is attrition among those who survived to 2023. Attrition analyses comparing those who were interviewed (56.4% of those who survived to 2023) with those who were living but not interviewed at age 62 reveal few significant differences on a wide range of childhood characteristics. However, compared to those who were living but not interviewed at age 62, the analytic sample is less likely to have a criminal history by age 42 (i.e., arrest and/or incarceration), more likely to be female, have higher levels of education, less likely to be poor in childhood and/or adolescence, and more likely to self-report good, very good, or excellent health at age 42. Because item missingness was minimal,^4^ we employ central tendency imputation as appropriate (i.e., mean, median, or mode) and conduct complete case analyses on the full age 62 sample.

### Measures

#### Incarceration History

*Incarceration exposure* is a three-category variable based on two self-reported questions from the age 62 interview: Have you ever been arrested, and Have you ever spent time in prison or jail by age 62?, coded as 0 = never arrested, 1 = arrested but never incarcerated, and 2 = incarcerated.^5^ In the interviewed sample, 62% were never arrested, 15% were arrested but never incarcerated, and 23% were incarcerated at least once by age 62. It is important to note that the vast majority of those with an incarceration history were living in the community at the time of the interview with an average of 20 years since their last incarceration.^6^ Five cohort members were interviewed in prison at age 62.

*Severity of incarceration history* is based on a combination of the number of self-reported incarcerations by age 62 [mean (sd): 4.56 (14.38); range: 1 to 150] and self-reports of the longest amount of time spent incarcerated in days by age 62 [mean (sd): 773.65 (1719.70); range: 1 to 14,610]. For the number of incarcerations, we created a dichotomous measure among those incarcerated (*n* = 121) of one incarceration (40.5%) and more than one incarceration (2 or more; 59.5%). For duration length, we created a dichotomous measure of less than or equal to one year as the longest incarceration stint (62.8%) and longer than one year as the longest stint (37.2%).

Finally, we operationalize *severity of incarceration history* as a four-category measure comparing one short incarceration (32.2%; mean length = 26.3 days), one long incarceration (8.3%; mean length = 1416.1 days), more than one but all short incarcerations (30.6%; mean number = 8.61; mean length = 66.2 days), and more than one incarceration with at least one long incarceration (28.9%; mean number = 5.25; mean length = 2170.7 days). These dichotomization decisions allow our interpretation to roughly align with comparing those with one incarceration versus multiple incarcerations and, in the US context, those spending time only in jail settings versus at least one experience in a prison setting (based on length of incarceration). This distinction becomes meaningful so far as it equates to different health-related experiences and social service programming. For instance, in the United States, those incarcerated for more than a year, typically in prisons, have more comprehensive health care, perhaps being diagnosed and treated for medical conditions for the first time (Schnittker et al., 2022), as opposed to those incarcerated for less than a year, typically in jail settings, where fewer health services are available (Turney & Conner, 2019).

#### Domains of Healthy Aging

Drawing from extensive reviews of the domains and measures used in studies of healthy aging (e.g., Lu et al., 2019; Zanjari et al., 2017; Michel & Sadana, 2017; Zamudio-Rodríguez et al., 2021), we operationalize healthy aging as an index of 10 indicators. We determined whether a participant was “healthily” aging or not for each indicator based on an absence of disease (e.g., physical health conditions), non-compromised status (e.g., memory and hearing), or clinical cutoffs (e.g., sleep, cognitive functioning). We then summed the 10 dichotomous indicators into an index of healthy aging ranging from 0 to 10. Table 1 details each domain, original indicator, and our recoded dichotomous measure of healthy aging for each indicator.

**Table 1:** Measures of Healthy Aging (n=531)

Domain	Measure (range)	Mean (sd)/ Percent	Definition of Healthy Aging	% Healthy Aging
Physical/ Physiological Health	Physical Health Conditions (0–3)	1.49 (1.12)	Has no physical health conditions	24.1%
	Functional Impairment from Physical Health (0–1)	36.2%	Has no physical health impairment	63.8%
	Daily Interference from Pain (1–5)	2.31 (1.45)	No interference from pain (rating = 1)	45.0%
Psychological/ Social Health	Mental Health Conditions (0–3)	0.48 (0.80)	Has no mental health conditions	66.3%
	Functional Impairment from Emotional Issues (0–1)	17.5%	Has no emotional impairment	82.5%
	Sleep Functioning (PSQI-SF) (0–15)	5.41 (2.96)	Normal sleep quality (score < = 4)	41.4%
	Loneliness (0–3)	1.02 (0.84)	Never/hardly ever lonely (score < = 1)	58.9%
Cognitive Health	Self-Rated Hearing (1–5)	3.79 (0.93)	Excellent hearing (rating = 5)	25.8%
	Self-Rated Memory (1–5)	3.43 (0.93)	Very good/excellent memory (rating > = 4)	48.4%
	Cognitive Functioning (TICS-M) (1–27)	13.42 (4.68)	Normal cognition (score > = 12)	64.6%

Exploratory factor analysis finds that the ten items listed in Table 1 load onto three factors (see Appendix B). *Physical/Physiological Health* captures one’s biological ability to prevent and recover from illness or disease as well as one’s ability to actively engage physically in all aspects of one’s life. One’s physical health is operationalized as the number of up to 13 current self-reported chronic physical health conditions,^7^ truncated at 3 or more conditions, with healthy aging defined as having no chronic physical conditions (i.e., absence of chronic disease).^8^ To capture physical functional capability, we use two past-month measures from the SF-12 health survey (Ware et al., 1996): (1) whether the participant accomplished less and/or was limited in their work because of physical health problems, and (2) how often pain interfered with daily activities, ranging from “not at all” (1) to “very, very much” (5). Healthy aging was defined as having no limitations from physical health and an absence of pain, respectively.

*Psychological/Social Health* captures both the absence of mental health conditions and the ability to actively engage mentally and socially in all aspects of one’s life. This domain is operationalized through (1) the number of current mental health conditions out of a possible three conditions (PTSD, anxiety, and depression^9^) where healthy aging is defined as having no mental health conditions; (2) whether the participant accomplished less and/or did their work or activities less carefully because of emotional problems based on two of the SF-12 health survey items (Ware et al., 1996), with healthy aging defined as no limitations from emotional problems; (3) sleep functioning, which is assessed using the Pittsburgh Sleep Quality Index-Short Form (PSQI-SF) scale that ranges from 0 to 15 where healthy sleep functioning is defined as scoring less than the clinical cutoff of five (Famodu et al., 2018); and (4) loneliness measured using the three-item UCLA Loneliness Scale (Hughes et al., 2004), which is a mean scale tapping into how often someone lacks companionship, feels left out, or feels isolated from others (range: never (0) to often (3)). Healthy aging on this indicator is defined as being never or hardly ever lonely (i.e., a score of 0 or 1) to capture healthy aging as the presence of meaningful social connections that are hallmarks of healthy aging (WHO, 2015).

*Cognitive Health* captures one’s functional capabilities with respect to hearing, memory, and the ability to process information. This domain is operationalized through three indicators – self-rated hearing and self-rated memory, both of which range from poor (1) to excellent (5), and cognitive functioning, which is a summed scale based on the Modified Telephone Interview Cognitive Status (TICS-M) scale, which ranges from 1 to 27 (Welsh et al., 1993). Drawing on research that indicates even mild hearing loss can have significant cognitive ramifications (Lin et al., 2013), we define healthy self-rated hearing as excellent and define healthy self-rated memory as very good or excellent to indicate non-compromised hearing and memory, respectively. We use the clinical cutoff of normal cognition (a score of 12 or greater) to define healthy cognitive functioning (Welsh et al., 1993).

#### Healthy Aging Index

Using these ten dichotomized indicators we create a summed index to capture a holistic concept of healthy aging (mean = 5.21, sd = 2.43). To establish the validity of our healthy aging index, we ran a series of correlations with measures of successful aging, which is a highly related but distinct concept. Whereas healthy aging emphasizes physical, mental, and cognitive capacity and capabilities, successful aging emphasizes life satisfaction and personal well-being (Wong, 2018). As evidence of its validity, our healthy aging index is significantly and moderately correlated with three measures of life satisfaction and well-being in older age. Those who score higher on the healthy aging index also 1) indicate higher levels of success in aging based on the question, “On a scale from 1 to 10, with 1 meaning ‘not well at all’ and 10 meaning ‘completely well,’ how successfully do you think you are aging” (r =.447, p <.001); 2) report higher values on the Attitude Toward Own Aging scale (Lawton, 1975), which is a mean score of eight items tapping into one’s attitudes about getting older (r =.555, p <.001); and 3) rate being happier based on the question, “If you were to consider your life in general these days, how happy or unhappy would you say you are, on the whole” with responses ranging from unhappy usually to extremely happy,” (*r* =.469, *p* <.001). Importantly, these correlations are far from perfect, providing evidence that one’s health and functional ability are related to but distinct from one’s perceptions of their success in the aging process.

#### Early Life Confounders

In order to examine the long-term influences of incarceration experiences on healthy aging while acknowledging the importance of early life experiences in shaping life’s trajectories (Elder, 1985), we draw on early life data to better isolate the associations between incarceration and aging, above and beyond early risk factors. Along with race, age, and early neighborhood context, which are held constant by design, we include eight covariates drawn from earlier assessments that tap into key correlates of incarceration risk and healthy aging. These covariates include: (1) *sex of the respondent* (female = 0, male = 1; 42.0%); (2) early life *poverty* that captures whether the household income was at or below the U.S. government poverty threshold based on family size in first grade and/or in adolescence (60.5%); (3) early life *residential instability* that represents the number of residential moves between birth and 1975 as reported by the mother or mother surrogate (mean = 2.19, sd = 1.66); (4) *high school non-completion* (0 = high school graduate or GED; 1 = high school non-completion; 17.3%), based on self-reports in young adulthood and Board of Education administrative data; (5) *low birthweight*, which is measured using 10 categories ranging from 0 = 8 pounds or more to 9 = less than 3.5 pounds (mean = 2.36, sd = 2.21); (6) mother’s reports of whether the child had a *chronic condition* at age 6 (3.8%); (7) teacher-reported *aggressive behavior* in first grade, defined as fighting too much, stealing, lying, resisting authority, damaging property, and being uncooperative, ranging from 0 (adaptation) to 3 (severe maladaptation) (mean = 0.47, sd = 0.86); and (8) *serious adolescent delinquency*, which is defined as being among the top 15% of the frequency distribution of 18 self-reported adolescent delinquent behaviors drawn from the adolescent interview or among the top 10% of the frequency distribution of nine self-reported adolescent delinquent behaviors prior to age 15 drawn from the young adult interview (14.9%) (see Doherty et al., 2008).

### Analytic Strategy

First, we assess the bivariate relationship between each individual indicator of healthy aging, as well as the healthy aging index measure and (1) incarceration exposure among the full sample of 531 participants or (2) severity of incarceration for the 121 participants who reported an incarceration history. We present the percentages reporting healthy aging and assess the statistical significance of these relationships using chi-square tests of independence for each dichotomous indicator and ANOVA for the healthy aging index measure. Second, we use generalized linear models to estimate the relationship between incarceration and the healthy aging index, controlling for early covariates.^10^ We report the regression coefficients and statistical significance for incarceration exposure using the never arrested as the reference category in Model 1 and incarceration as the reference category in Model 2. We then estimate models for severity of incarceration using one short incarceration as the reference category (Model 3) and more than one, at least one long incarceration stint as the reference category (Model 4). To aid in the interpretation of the results, we present the estimated marginal means of the healthy aging index across the exposure and severity categories. All analyses were conducted using SPSS.

## Results

### Descriptive Statistics on Healthy Aging

Table 1 highlights that limiting indicators to the presence or absence of health conditions provides a narrow picture of healthy aging. For example, although the majority of the sample is living with at least one chronic physical health condition (75.9%), 63.8% report no impairments from their physical health and 82.5% report no emotional impairments. However, many of the indicators show evidence of weathering (Geronimus, 2023) at age 62 for both incarcerated and non-incarcerated individuals. For instance, many of the participants have at least some hearing loss (74.2%) and do not sleep well, based on standard cutoffs of normal quality sleep (58.6%). The cognitive functioning mean score of 13.42 for the sample is comparable to similarly-aged Black participants (14.25), yet it is lower than estimates of white participants (16.12) from the Health and Retirement Study (Byrne & Anaraky, 2022).

### Bivariate Associations between Incarceration History and Healthy Aging

As a first step to examining whether healthy aging differs based on incarceration history, we compare the bivariate associations between each of the healthy aging indicators, as well as the healthy aging index, across the three incarceration exposure categories and the four severity of incarceration categories. Table 2 displays the percentages, test statistics, and p-values of the comparisons. Eight of the 10 indicators of healthy aging were significantly different across the incarceration exposure categories (*p* <.05). Examination of the percentages reveals a steady decline in healthy aging as the severity of criminal legal system experiences increases from never arrested to incarcerated for 3 of the 8 indicators that significantly differ across groups (i.e., hearing, memory, and mental health conditions); however, for 5 of the 8 indicators (i.e., pain, physical and emotional impairment, sleep, and cognitive functioning), the significant differences seem to be driven by the comparison between the never arrested group and having any level of criminal legal system involvement. Finally, the healthy aging index scores gradually decrease from higher levels of healthy aging among the never arrested group (mean = 5.67) to lower healthy aging scores among the incarcerated group (mean = 4.38) with post-hoc tests showing that the never arrested group significantly differs from the incarcerated group, but there is no significant difference between the arrested but not incarcerated group and the incarcerated group at *p* <.05.

Table 2 also shows that once the sample is restricted to those who have been incarcerated at least once by age 62, the combination of the number and length of the longest incarceration is only related to two of the healthy aging indicators - pain interference and mental health conditions. An examination of the percentages shows distinct patterns that do not follow a gradual decrease with severity of exposure. For instance, for pain interference, most of those with one long incarceration (8 of the 10 people in this group) report no pain interference compared to the other three groups, which have closer to 30 or 40 people in them and show 20% to 43% reporting no pain. For mental health conditions, those who have one short incarceration are more likely to be aging healthily than those who have more than one incarceration and at least one long (74.4% and 34.3% with no mental health conditions, respectively). Finally, the healthy aging index scores are comparable among the two groups with only one incarceration – 4.95 for those with a short stay and 5.20 for those with a long stay, which are higher than those with more than one incarceration. The lowest healthy aging score, on average, is among the group who experiences more than one incarceration and at least one long (mean = 3.54).

### Multivariable Regression Models

**Fig. 1:**
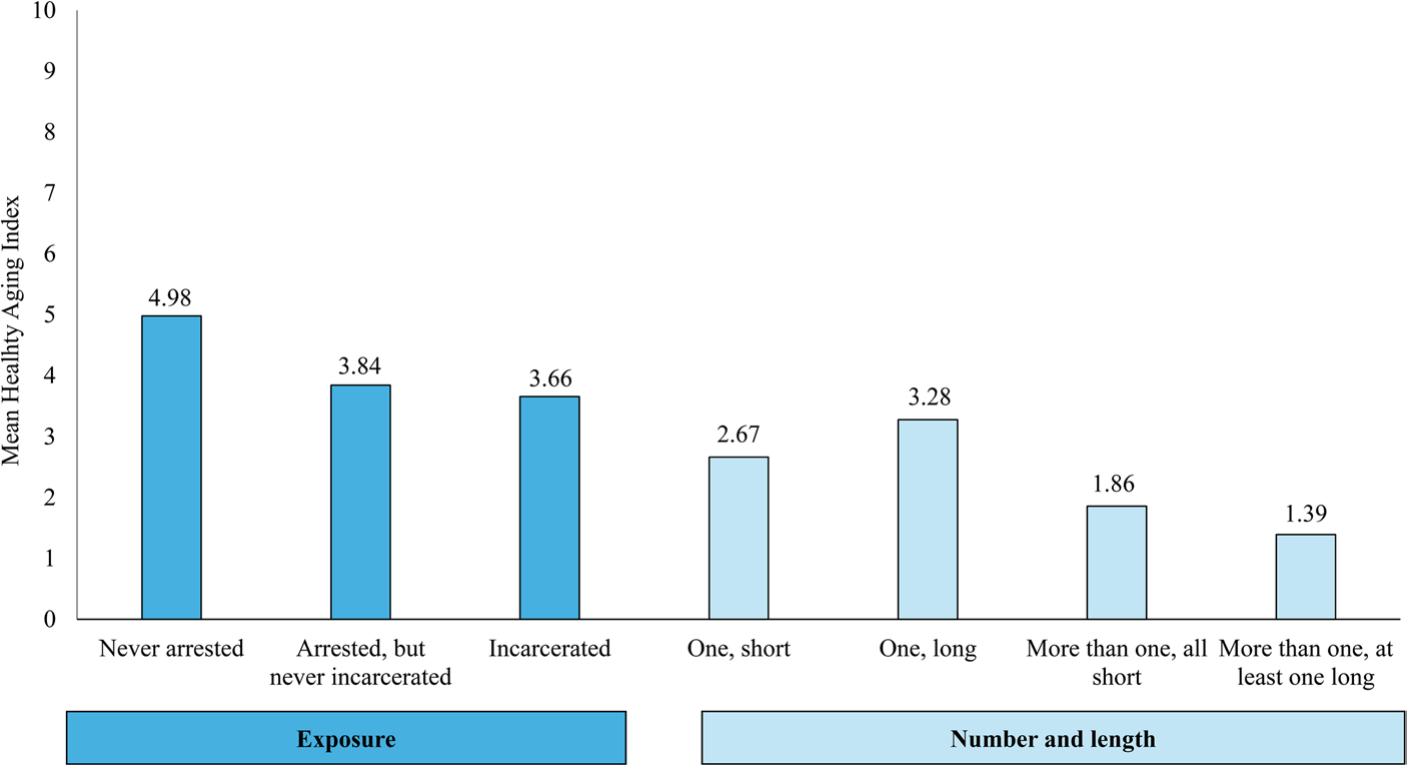
Predicted marginal means of healthy aging by incarceration exposure and severity

Using our global healthy aging index that captures the many facets of the aging experience holistically, we first test the hypothesis that incarceration exposure is related to healthy aging, controlling for key early life factors for the full sample (see Table 3, Models 1 and 2). Findings from Model 1 indicate that both the incarcerated group and arrested but never incarcerated group report significantly lower levels of healthy aging when compared to the never arrested group (b=−1.317 and − 1.139, respectively, *p* <.001). However, when the reference group is changed to compare the arrested but never incarcerated group to the incarcerated group, there is a positive but non-significant difference in healthy aging (b = 0.178, *p* =.603; Model 2). The estimated marginals means depicted on the left side of Fig. 1 show that the never arrested group has a predicted mean healthy aging score of 4.98, which is significantly higher than the mean scores of 3.84 and 3.66 of the arrested but never incarcerated group and incarcerated group, respectively.

**Table 2:** Generalized linear regression estimates of healthy aging index on incarceration history

		Full Sample (*n* = 531)				Incarcerated Sample (*n* = 121)				
		Never Arrested (*n* = 329)	Arrested, Never Incarcerated (*n* = 81)	Incarcerated (*n* = 121)	X^2^ (*p*-value)	One and Short (*n* = 39)	One but Long (*n* = 10)	More than one but all short (*n* = 37)	More than one and at least one long (*n* = 35)	X^2^ (*p*-value)
Physical/ Physiological Health	No physical health conditions	24.0%	28.4%	21.5%	NS	20.5%	50.0%	16.2%	20.0%	NS
	No physical health impairment	69.9%	53.1%	54.5%	13.835 (*p* <.001)	56.4%	50.0%	64.9%	42.9%	NS
	No pain interference	50.2%	35.8%	37.2%	9.278 (*p* =.010)	35.9%	80.0%	43.2%	20.0%	12.882 (*p* =.005)
Psychological/Social Health	No mental health conditions	72.0%	60.5%	54.5%	13.548 (*p* =.001)	74.4%	50.0%	54.1%	34.3%	12.056 (*p* =.007)
	No emotional impairment	87.2%	71.6%	76.9%	14.424 (*p* <.001)	84.6%	90.0%	78.4%	62.9%	NS
	Normal sleep quality	45.6%	33.3%	35.5%	6.269 (*p* =.044)	38.5%	40.0%	35.1%	31.4%	NS
	Low loneliness	62.6%	51.9%	53.7%	NS	64.1%	50.0%	54.1%	42.9%	NS
Cognitive Health	Excellent hearing	30.1%	21.0%	17.4%	8.652 (*p* =.013)	17.9%	20.0%	16.2%	17.1%	NS
	Very good/excellent memory	54.7%	43.2%	34.7%	15.200 (*p* =.001)	41.0%	50.0%	24.3%	34.3%	NS
	Normal cognition	71.1%	56.8%	52.1%	16.596 (*p* <.001)	61.5%	40.0%	48.6%	48.6%	NS
Mean Healthy Aging Index (sd)		5.67 (2.31) Range: 1–10	4.56 (2.56) Range: 0–9	4.38 (2.37) Range: 0–9	F = 16.963 (*p* <.001)	4.95 (2.18) Range: 1–9	5.20 (2.86) Range: 0–9	4.35 (2.29) Range: 0–9	3.54 (2.34) Range: 0–8	F = 2.723 (*p* =.047)

**Table 3:** Generalized linear regression estimates of healthy aging index on incarceration history

	Model 1 Full Sample (*n* = 531)		Model 2 Full Sample (*n* = 531)		Model 3 Incarcerated Sample (*n* = 121)		Model 4 Incarcerated Sample (*n* = 121)	
	coeff (se)	*p*-value	coeff (se)	*p*-value	coeff (se)	*p*-value	coeff (se)	*p*-value
Constant	5.787 (0.246)	< 0.001	3.060 (0.686)	< 0.001	5.836 (0.720)	< 0.001	−0.735 (1.60))	0.646
Full Sample (*n* = 531)								
Never Arrested	ref.		**1.139 (0.297)**	**< 0.001**	---	---	---	---
Arrested, but Never Incarcerated	**−1.317 (0.284)**	**< 0.001**	0.178 (0.342)	0.603	---	---	---	---
Incarcerated	**−1.139 (0.297)**	**< 0.001**	ref.		---	---	---	---
Among Incarcerated (*n* = 121)								
One and short	---	---	---	---	ref.	----	**1.272 (0.521)**	**0.015**
One but long	---	---	---	---	0.616 (0.776)	0.428	**1.887 (0.809)**	**0.020**
More than one, all short	---	---	---	---	− 0.806 (0.500)	0.107	0.466 (0.515)	0.366
More than one, at least one long	---	---	---	---	**−1.272 (0.521)**	**0.015**	ref.	----
Childhood and Adolescent Controls								
Sex (1 = male)	**0.676 (0.231)**	**0.003**	**0.676 (0.231)**	**0.003**	**1.030 (0.479)**	**0.031**	**1.030 (0.479)**	**0.031**
Early Poverty	−0.073 (0.212)	0.732	−0.073 (0.212)	0.732	**−1.201 (0.465)**	**0.010**	**−1.201 (0.465)**	**0.010**
Residential Instability	− 0.066 (0.064)	0.301	− 0.066 (0.064)	0.301	− 0.092 (0.135)	0.498	− 0.092 (0.135)	0.498
High School Non-completion	**−0.781 (0.279)**	**0.005**	**−0.781 (0.279)**	**0.005**	0.016 (0.429)	0.970	0.016 (0.429)	0.970
Low Birthweight	0.028 (0.047)	0.549	0.028 (0.047)	0.549	− 0.068 (0.094)	0.471	− 0.068 (0.094)	0.471
Childhood Chronic Condition	− 0.699 (0.537)	0.193	− 0.699 (0.537)	0.193	**−4.471 (1.369)**	**0.001**	**−4.471 (1.369)**	**0.001**
First Grade Aggressive Behavior	− 0.051 (0.122)	0.675	− 0.051 (0.122)	0.675	− 0.195 (0.224)	0.385	− 0.195 (0.224)	0.385
Serious Delinquent (Adolescence)	− 0.533 (0.292)	0.068	− 0.533 (0.292)	0.068	− 0.674 (456)	0.139	− 0.674 (456)	0.139

**Bold =** *p* <.05

The models for the ever-incarcerated subsample (Table 3, Models 3 and 4), show the results regarding the relationship between the severity of incarceration and the healthy aging index. We find that having the combination of more than one incarceration where at least one was long is significantly related to lower healthy aging scores than having one short incarceration (b=−1.272, *p* =.015). When the reference group is changed to the more than one and at least one long incarceration group, there is also a statistically significant difference in healthy aging between that group and the one long incarceration group (b = 1.887, *p* =.020). This relationship is shown on the right side of Fig. 1 where the mean healthy aging score is lower for the more than one incarceration group (1.39) compared with either of the groups experiencing only one incarceration (2.67 and 3.28 for one short and one long, respectively).

## Discussion

This study’s primary purpose was to expand scholarship on the life course consequences of incarceration to include the concept of healthy aging. The conceptualization of healthy aging used in this study draws on several domains that encompass not only the presence or absence of disease but also the capacity and functional ability in older age that together represent healthy aging. Moreover, by using comparison groups that distinguish incarcerated individuals from those arrested with no incarceration, we sought to determine the consequences of incarceration that are distinct from earlier stages of criminal legal system involvement.

Our findings show moderate levels of healthy aging for the sample as a whole at age 62 with a mean just over 5 out of 10 on the index and that those who are incarcerated age into the early 60s less healthily than those who were never arrested. However, those who were arrested without incarceration also experienced less healthy aging than the never arrested and there was no significant difference in healthy aging between the arrested and incarcerated groups. That is, while incarceration is associated with less healthy aging overall and across many of the individual indicators, many detriments on aging are equally felt among those whose experiences are limited to earlier stages of criminal legal system contact (i.e., those who are arrested but not incarcerated). This finding was somewhat unexpected and warrants future research to better understand the mechanisms underpinning how all stages of contact (e.g., stops and search, arrest, conviction) are related to (un)healthy aging, including the accumulation of experiences along the continuum (e.g., LeMasters et al., 2022). These investigations are particularly critical for health disparities work given that all types of criminal legal system contact are disproportionately experienced by Black Americans (National Academy of Sciences, 2023).

The findings from this largely cross-sectional study represent a first step in building scholarship on the association between criminal legal system contact and healthy aging from a life course perspective and sets the stage for future life course research that align with its key principles of human agency, linked lives, timing, and historical context (Elder, 1998). For instance, one key domain that underpins healthy aging and is “essential to enable older people to do the things that they value” (WHO, 2015, p. 159) is the ability to meet one’s basic needs through financial security, housing, and personal safety. Thus, future research should focus on disentangling the relationships between the array of criminal legal system contact types (e.g., police stop without arrest, arrest without conviction, conviction without incarceration, etc.) that garner different financial consequences, such as limits on housing and employment opportunities, and healthy aging (Beckett & Goldberg, 2022; Travis et al., 2014; Sampson & Laub, 1997; Pager, 2008; Evans et al., 2019; Stewart & Uggen, 2020; DeMarco et al., 2021; DeMarco, 2024). This future research direction would benefit from drawing on the concept of human agency and situated choice as the connection between criminal legal system involvement and compromised aging may be through its impact on how these cumulative disadvantages constrain one’s aging-related opportunities and decisions as one navigates the aging process.^11^

Another key domain of healthy aging is the ability to build and maintain relationships in older age (WHO, 2015), which is closely related to the life course principle of linked lives. Future research should examine how incarceration might indirectly affect healthy aging and the aging process through the severance of social bonds and networks as a consequence of criminal legal system involvement (e.g., Beckett & Goldberg, 2022; Sampson & Laub, 1997) that then compromises one’s ability to build and maintain relationships in later life. Alternatively, research should examine how one’s ability to garner social support, even among limited social networks, might buffer the relationship between incarceration and healthy aging.^12^

Third, we were not able to study the associations between the timing of incarceration and healthy aging at 62, which could be an important direction for future research. Similar to research on the timing of salient positive life events, such as work or marriage on desistance (Uggen, 2000; Theobald & Farrington, 2011), future research could examine how the potential immediate consequences of incarceration upon reentry (e.g., financial insecurity, social relationships) differ in their effect on the aging process and healthy aging depending on when one’s first and last incarceration stint occur within the development of the life course (e.g., young adulthood vs. older age). These types of questions are particularly relevant in light of decarceration efforts in the U.S. (Clear, 2021).

Fourth, future research should focus on how historical and cultural context shape aging norms and the meanings people ascribe to age (i.e., subjective aging) as these are not only grounded in context but also shaped by socialization experiences, including criminal legal system experiences (Settersten and Hagastad, 2015). For instance, Reich and colleagues (2020) conducted a systematic review of what successful aging meant to people of different races and ethnicities and found that for Black/African Americans, social engagement (particularly family), independence, and physical health were paramount; other studies found that Black adults often describe themselves as healthy despite having chronic health conditions (Troutman et al., 2011; Griffith et al., 2018). Thus, against the backdrop of pervasive health disparities and premature mortality that disproportionately expose Black Americans to disability and deaths of household members and family (Dixon 2024; Umberson et al., 2017), it could be that the meaning assigned to “healthy” aging among Black Americans who survive to age 62 is defined by their survivorship as these individuals have “weathered” the storms and have shown great resilience compared to other Black Americans (Geronimus, 2023). Indeed, “weathering” incarceration stints and reintegrating into one’s community successfully across a variety of domains has been championed as the preferred hallmarks of success in the reentry context (National Academy of Science, 2022).

Finally, although this study focused on a highly relevant population of Black Americans in their 60s, future research should examine these relationships for younger and/or older samples to capture the full aging process as well as across gender, race, and ethnicity within the US setting. Moreover, given the unique context of the US criminal legal system, our understanding of the relationship between incarceration and healthy aging would be elevated by research from a variety of international contexts that differ from the US context on key dimensions such as lower incarceration rates, significantly shorter sentences, and comprehensive social safety nets both inside and outside the carceral environment, including healthcare provisions (e.g., Mauer, 2017; Weiss et al., 2020).

In conclusion, as the age distribution of the US population shifts to older ages, it is important to understand how life experiences, such as criminal legal system encounters and experiences, shape one’s trajectory of healthy aging into later life. Knowing that those who have incarceration histories are more likely to have compromised health conditions prior to incarceration (Turney and Conner, 2019; Schnittker et al., 2022), it is imperative to better understand how these early life trajectories intersect with one’s economic and social trajectories that contribute to the (re)direction of one’s aging trajectories, more broadly defined, to fully inform our understanding of criminal legal system involvement and the aging process across the full life course.

## Supplementary Information

Below is the link to the electronic supplementary material.

Supplementary File 1 (DOCX 22.6 KB)

Supplementary File 2 (DOCX 14.9 KB)

## Footnotes

1 To be clear, in this study we use the arrest but no incarceration group as one of the comparison groups to examine our primary research question, which focuses on the relationship between incarceration and healthy aging. Thus, we do not hypothesize or theorize about the relationship between arrest in the absence of incarceration and healthy aging in this study.

2 Mortality data, which include deaths through 2023, are drawn from the National Death Index (NDI) following a robust protocol for assessing mortality. NDI matches were made using demographic information (first, middle, and last name; sex; race; date of birth; birth state; residential state), as well as Social Security numbers, which were available for the majority of participants. Over the past 25 years, death certificates were also obtained for verification when no NDI match was found but a family member or public record indicated a Woodlawn cohort member was deceased.

3 Incarceration histories were self-reported at either the young adult or midlife interview and arrest data was drawn from official arrest records (see Doherty & Ensminger, 2014).

4 The majority (18 measures) of the 21 measures used in the analysis had less than 3% missing, one measure was missing less than 5% (high school non-completion), and two measures were missing 7% (sleep functioning and adolescent delinquency).

5 Self-report data on one’s incarceration history is subject to recall bias, particularly if it is differentially related to poor cognitive health. Using the Woodlawn prospective data, we find considerable overlap between earlier reports of incarceration from the age 32 and/or age 42 assessments and the age 62 reports. For instance, of the 333 cohort members who said that they had never been incarcerated by age 62, 7% had previously reported being incarcerated in a previous wave. However, recanting does not seem to be related to key health indicators, including cognitive functioning (data available upon request).

6 The interview asked about the ages of incarceration for those who reported having been incarcerated by age 62. Although the data was usable for only 91 out of the 121 participants who reported incarceration, these data indicate an average age of 40.22 as the age of last incarceration, with a range of 16 to 62; 75% were last incarcerated at age 50 or younger.

7 These 13 chronic conditions are primarily conditions directly related to stress (e.g., heart disease, high blood pressure/hypertension) or are conditions that are exacerbated by stress (e.g., diabetes, autoimmune diseases), which have been more strongly related to incarceration history (Massoglia, 2008). The most common chronic conditions reported by the age 62 sample are high blood pressure/hypertension (59%), high cholesterol (35%), and diabetes (27%).

8 The study did not collect biomarker data (e.g., blood, saliva) to identify the presence of physical health conditions. Because self-report measures of chronic physical health conditions require that the participant know about their condition, this measure likely undercounts participants with conditions with low symptomatology, those at the pre-disease stage, or those who lack access to quality medical care.

9 A participant was coded as reporting depression at age 62 if they answered “yes” to whether they had a depressive disorder, such as depression or bipolar, or if they scored 10 or higher on the short form of the CES-D scale (Shrout & Yager, 1989), indicating significant depression symptoms. The mean number of mental health conditions among those reporting at least one is 1.43 with the vast majority of those reporting a depressive disorder or meeting the cutoff for significant depression symptoms (93.7%) with fewer reporting PTSD (21.4%) or anxiety (30.3%).

10 We use generalized linear regression as opposed to ordinary linear regression to allow for the slightly negative skewed distribution of the healthy aging index measure.

11 To examine the fruitfulness of this line of inquiry, we ran chi-square tests of independence among the Woodlawn Age 62 sample and found a clear relationship between arrest, incarceration, and (1) financial insecurity (e.g., 49.5% among the never arrested, 25.9% among the arrested, and 29.8% among the incarcerated had both an individual retirement account and savings account; *X*^2^ = 46.718, *p* <.001) and (2) housing insecurity (e.g., 55.6% among the never arrested, 42.0% among the arrested, and 27.3% among the incarcerated owned their own home at age 62; *X*^2^ = 29.538, *p* <.001).

12 To examine the fruitfulness of this line of inquiry, we ran an ANOVA among the Woodlawn Age 62 sample and found a significant relationship between the three incarceration exposure groups and social support on the seven-item PROMIS social support scale (Rung Wu et al., 2024), which is a mean scale ranging from 0 to 3. Mean scores for the incarcerated, arrested, and never arrested groups are 2.22, 2.45, and 2.50, respectively (F = 8.794, *p* <.001).
