# Supplementary material for: Examining the Relationship Between Incarceration and Healthy Aging
Source: J Dev Life Course Criminol. 2025 Dec 3;11(1-4):345–66. doi: 10.1007/s40865-025-00286-5 (PMC12756256; doi:10.1007/s40865-025-00286-5)
Supplement: Supplementary file 1 — (DOCX 22.6 KB) [file 40865_2025_286_MOESM1_ESM.docx]

Appendix A: Relationship between Incarceration History by Age 42 and Mortality (n=944)

Timing of Death by Arrest and Incarceration History by Age 42

The Kaplan-Meier curves show that those incarcerated by age 42 were significantly more likely to have died by 2023 than those who had never been arrested or incarcerated by age 42 (24.0% and 11.8%, respectively), but that this group was not more likely to die by 2023 than those who were arrested but not incarcerated (22.6%) based on the Mantel-Cox log rank test (*X*^2^ = 21.402, p<.001).

Cox Proportional Hazards Regression Models for Mortality Risk by Arrest and Incarceration History by Age 42

|  | **Model 1: Unadjusted Hazard Ratio (n=944)** | ***p*-value** | **Model 2: Adjusted Hazard Ratio (n=944)** | ***p*-value** |
| --- | --- | --- | --- | --- |
| Never arrested by age 42 | ref. | --- | ref. | --- |
| Arrested but not Incarcerated by age 42 | 2.008 | <.001 | 1.865 | .004 |
| Incarcerated by age 42 | 2.166 | <.001 | 1.784 | .016 |
| Sex (1=male) | --- | --- | 0.991 | .960 |
| Early Poverty | --- | --- | 1.397 | .074 |
| Residential Instability | --- | --- | 0.932 | .185 |
| High School Non-completion | --- | --- | 1.738 | .004 |
| Low Birthweight | --- | --- | 1.017 | .629 |
| Childhood Chronic Condition | --- | --- | 2.382 | .013 |
| First Grade Aggressive Behavior | --- | --- | 1.085 | .356 |
| Serious Adolescent Delinquent | --- | --- | 1.145 | .522 |

The Cox regression models estimate unadjusted and adjusted hazard ratios, controlling for several early life confounders described in the Measures section. These models reveal that an incarceration history is associated with more than 1.7 times the mortality risk of those never arrested (aHR = 1.784, *p* = .016), with a similarly higher risk among those who were arrested but not incarcerated (aHR = 1.865, *p* = .004).
