# Supplementary material for: Examining the Relationship Between Incarceration and Healthy Aging
Source: J Dev Life Course Criminol. 2025 Dec 3;11(1-4):345–66. doi: 10.1007/s40865-025-00286-5 (PMC12756256; doi:10.1007/s40865-025-00286-5)
Supplement: Supplementary file 2 — (DOCX 14.9 KB) [file 40865_2025_286_MOESM2_ESM.docx]

Appendix B: Exploratory Factor Analysis, Rotated Solution (n=531)

| **Domain** | **Measure** | **Factor Loadings**  **1 2 3** | | |
| --- | --- | --- | --- | --- |
| **Physical/**  **Physiological Health** | Physical Impairment | **0.765** | 0.201 | -0.043 |
|  | Pain interference | **0.751** | 0.289 | -0.118 |
|  | PH Conditions | **0.673** | 0.035 | -0.125 |
| **Psychological/**  **Social Health** | MH Impairment | 0.199 | **0.767** | -0.029 |
|  | MH Conditions | 0.256 | **0.761** | -0.117 |
|  | Loneliness | -0.020 | **0.726** | -0.204 |
|  | Sleep | 0.353 | **0.519** | -0.209 |
| **Cognitive Health** | Memory | -0.155 | -0.148 | **0.743** |
|  | Hearing | -0.154 | -0.118 | **0.723** |
|  | Cognition | 0.002 | -0.088 | **0.601** |
